# Supplementary material for: Chromothripsis during telomere crisis is independent of NHEJ, and consistent with a replicative origin
Source: Genome Res. 2019 May;29(5):737–49. doi: 10.1101/gr.240705.118 (PMC6499312; doi:10.1101/gr.240705.118)
Supplement: Supplemental Material [file supp_gr.240705.118_Supplemental_file_1.zip › contigs/annotated_contigs/DB78/contig.2.DB78_length_477_mean_cov_7.90566037736.docx]

**DB78_length_477_mean_cov_7.90566037736**

```
TGAGGTGGGTGGATTTCCTGAGCTCAGGAGTTTGAGACCAGCCTGGGCAACATGGTGAAACCCCATCTCTACTAAAATACAAAAAATTA
 >chr16:50067539-50067816 + E=6e-148 p=3e-03
GCCAGGTGTGGTGGTCTGTGCCTGTAGTCCCAGCTACTCGGGAGGCTGAGGTGGGAGAATCGCTTGAACCCGGGAGGCAGAGGTTGCAG

TGAGCTGAAATCACGCCACTGCACTCCAGCCTGGGCGACAGAGCAAGACTGCGTCTCAAAAAAAAAAAGAGTACGCTTTGTGTTTTTTT

TTTGAGA|TGATTTTTGAGATTTTTG|AGATGATTTTAAAGAACAGATGAGAAAAGTACATTCAGAAAAATGAGAGAAGGCTCATCCAC
                          >chr16:49998230-49998415 + E=9e-100
AGTTAGTTCCCAGGACAGACCCATACTTGTGGTCGTTACCACCAAGATGATTCCTTGCTAAAGAGTAAAAGAATGAGCTCATGCTGTCC

TCTCAACACTTTGGGAGGCCGAGATGGGAAGATC
```
